# Building prognostic models for breast cancer patients using clinical variables and hundreds of gene expression signatures

**DOI:** 10.1186/1755-8794-4-3

**Published:** 2011-01-09

**Authors:** Cheng Fan, Aleix Prat, Joel S Parker, Yufeng Liu, Lisa A Carey, Melissa A Troester, Charles M Perou

**Affiliations:** 1 Lineberger Comprehensive Cancer Center, University of North Carolina, Chapel Hill, USA; 2 Department of Genetics, University of North Carolina, Chapel Hill, USA; 3 Department of Statistics & Operations Research, University of North Carolina, Chapel Hill, USA; 4 Department of Medicine, Division of Oncology, University of North Carolina, Chapel Hill, USA; 5 Department of Epidemiology, University of North Carolina, Chapel Hill, USA; 6 Department of Pathology & Laboratory Medicine, University of North Carolina, Chapel Hill, USA; 7 Carolina Center for Genome Sciences, University of North Carolina, Chapel Hill, USA

## Abstract

**Background:**

Multiple breast cancer gene expression profiles have been developed that appear to provide similar abilities to predict outcome and may outperform clinical-pathologic criteria; however, the extent to which seemingly disparate profiles provide additive prognostic information is not known, nor do we know whether prognostic profiles perform equally across clinically defined breast cancer subtypes. We evaluated whether combining the prognostic powers of standard breast cancer clinical variables with a large set of gene expression signatures could improve on our ability to predict patient outcomes.

**Methods:**

Using clinical-pathological variables and a collection of 323 gene expression "modules", including 115 previously published signatures, we build multivariate Cox proportional hazards models using a dataset of 550 node-negative systemically untreated breast cancer patients. Models predictive of pathological complete response (pCR) to neoadjuvant chemotherapy were also built using this approach.

**Results:**

We identified statistically significant prognostic models for relapse-free survival (RFS) at 7 years for the entire population, and for the subgroups of patients with ER-positive, or Luminal tumors. Furthermore, we found that combined models that included both clinical and genomic parameters improved prognostication compared with models with either clinical or genomic variables alone. Finally, we were able to build statistically significant combined models for pathological complete response (pCR) predictions for the entire population.

**Conclusions:**

Integration of gene expression signatures and clinical-pathological factors is an improved method over either variable type alone. Highly prognostic models could be created when using all patients, and for the subset of patients with lymph node-negative and ER-positive breast cancers. Other variables beyond gene expression and clinical-pathological variables, like gene mutation status or DNA copy number changes, will be needed to build robust prognostic models for ER-negative breast cancer patients. This combined clinical and genomics model approach can also be used to build predictors of therapy responsiveness, and could ultimately be applied to other tumor types.

## Background

Genomic profiles have significantly improved our ability to prognosticate in breast cancer patients [1,2]. Several of these genomic predictors such as the NKI 70-gene signature (Mammaprint, Agendia) [3,4] or the OncotypeDX Recurrence Score (RS, Genomic Health) [5] are commercially available and commonly used. We and others have shown that these and other prognostic gene expression profiles are, in fact, similar in terms of outcome predictions despite a lack of gene overlap, suggesting that they each track a common set of biologic characteristics [6,7]. Since then, numerous signatures with the potential for increasing prognostic accuracy have been reported. Some of these have been developed to track activated molecular signalling pathways [8-19] and/or particular biological processes such as cell proliferation [17,18,20-22], hypoxia [23-26], cell differentiation [27-30], immune cell processes [27,31,32] and wound responses [33-36]; other signatures have been specifically designed to predict sensitivity to chemotherapy [37,38] or biologic therapies [39,40].

Many studies have examined the prognostic significance of genomic biomarkers along with clinical-pathological variables and often shown that both provide independent information [41-44]; however, very few studies have attempted to create integrated prognostic models that contain both genomic and clinical biomarkers [45]. We have recently shown that integration of one pathological variable (i.e. tumor size) with one genomic signature (i.e. intrinsic subtypes) outperforms either strategy alone in terms of outcome prediction [46], suggesting that both data types can provide independent prognostic power and be combined into a single model. With the explosion of signatures developed with distinct biologic processes in mind, it makes sense to take this approach one step further and develop models that include not only clinical and genomic information, but systematically examines inclusion of multiple genomic signatures in an effort to further hone prognostication beyond one profile versus another.

In this study, we developed a prognostic model in systematically untreated node-negative breast cancer patients derived from multiple commonly used clinical variables and a large database of gene expression modules, and we confirmed that models that incorporate both clinical and genomic variables are the most accurate for outcome predictions for newly diagnosed patients with node-negative breast cancer. Importantly, this approach to model development can also be used to predict responsiveness to therapy and could be generalized to other tumor types.

## Methods

### Patient Populations

A single large dataset of homogenously treated patients was created by combining 5 different publicly available clinically-annotated microarray datasets of node-negative breast cancer patients treated with local therapy only, and no adjuvant systemic therapy: van de Vijver et al.[4], Wang et al.[47], Loi et al.[48], Ivshina et al.[49], and the University of North Carolina (UNC) [13,14,50], where 4 new patients were included (GEO accession number of the UNC data, GSE15393). The gene expression data was combined using the batch effect adjustment by the Distance Weighted Discrimination (DWD) method [51].

Of the 666 patients included in this combined dataset, 550 (83%) had complete data on relapse-free survival (RFS, defined as the time to first relapse [local or distant]) and clinical variables (tumor size, histological grade, and estrogen receptor [ER]), and were included for further analyses using a 7-year cut-point for RFS. For the HER2 "clinical status", which was not available for most studies, we used a gene expression surrogate based on the mRNA levels of the HER2 gene, using the top 20% rank order highest expressers as the cutoff value for calling a tumor "HER2-positive"; the chosen cutoff value for HER2 positivity was based on a population-based study of breast cancer[52].

We examined the ability of this approach to predict pathological complete response (pCR) rate to neoadjuvant chemotherapy in a dataset from Popovici et al. (MDACC)[37,53], which includes 225 pre-treated samples/patients (MDACC225) that received neoadjuvant anthracycline/taxane-based chemotherapy and had complete clinical data. pCR was defined as the absence of invasive cancer in the breast and axillary lymph nodes.

### Gene expression Modules

We defined modules as sets of co-expressed genes that were considered as a functional unit. Using multiple approaches, we built a collection of 323 gene expression modules, including 115 gene lists obtained from 53 publications: (**1**) 221 modules were built using the median expression of all genes within the module that homogeneously expresses these genes (i.e. all genes in the module were high or low together within a given sample). The sources of the selected homogenous gene lists were the following: 50 were identified by bicluster analyses[54] using the microarray dataset of 359 human breast tumors and 8 normal breast samples (i.e. the aforementioned 2/3 training set); 52 modules were identified from an unsupervised hierarchical clustering analysis of the same human breast tumor database; 50 were identified by bicluster analyses using microarray data of 122 mouse mammary tumors[13]; 56 were identified from unsupervised hierarchical clustering analysis of the same mouse mammary database; 13 were identified from previously published gene lists [13,14,17,18,21,26,35,55]. (**2**) 77 modules were represented as the first Principal Component of previously published gene lists [3,8-10,12,13,15,19,20,22,23,25,27-32,34,36,37,40,56-66] that showed heterogeneous expression patterns (i.e. the gene list contained genes with high and low expression within a given sample). (**3**) 22 modules were correlations to previously published training dataset centroids [4,9,11,16,24,33,38,39,50,67,68]. (**4**) 3 modules were built from previously published gene expression prognostic models [5,46,47]. We acknowledge that our implementation of some of the previously published signatures may be suboptimal, however, we attempted within reason, to apply each signature as published. All modules, with gene lists and references, can be found in Additional File 1.

### Statistical Analysis

The various modules and clinical variables (tumor size, histological grade, ER and HER2 status) were evaluated by the Least Absolute Shrinkage and Selection Operator (LASSO) method to build prognostic models using a Cox proportional hazards approach[69]. For all analyses, a training set (~2/3) stratified by data source, platform and clinical variables was used to derive the modules and build a model, which was then applied to the testing set (~1/3) (10-fold cross-validation). We defined "success" of a model when prognostic significance for RFS (p < 0.05 by the Cox Model) was shown on both the training and testing sets. Survival curves were analyzed and compared with the use of the Kaplan-Meier method and the log-rank testing, and hazard ratios were derived from the Cox proportional hazards model. For the Kaplan-Meier analyses, patients were stratified into high and low-risk groups based on their respective risk score, which was defined as the natural logarithm of the hazard ratio with a chosen cut-off value for stratification into high and low-risk groups of zero. To further examine the most frequently selected modules and/or clinical variables that build successful prognostic combined models, we repeated 200 times the previous combined model-building procedure (i.e. by randomly splitting the training and testing sets), and then we calculated the frequency of selection of each module/clinical variable among these 200 successful models. Statistically over-represented (p < 0.001) biological processes within modules were identified with EASE http://david.abcc.ncifcrf.gov/.

The prognostic ability of the reproducible models and known prognostic predictors was further characterized by calculating the concordance index (C-index) [70]. The C-index is a measure of the probability that, given two randomly selected patients, the patient with the worse outcome is, in fact, predicted to have a worse outcome. This measure is similar to an area under the receiver operating characteristic curve, ranging from 0.5 to 1. For each cohort, we used the model built from the training set to calculate the C-index of the testing set. We repeated this procedure 200 times by randomly splitting the training and testing sets, and then we calculated the mean of the C-indexes of the 200 testing sets evaluated. Because they so strongly influenced prognostic models, and in order to illuminate other relevant signatures and biologic processes, we repeated the same analyses after excluding the NKI 70-gene signature [3,4], the OncotypeDX RS [5], the Rotterdam 76-gene signature [47] and the recently described Risk of Relapse (ROR) score based on the intrinsic subtypes (ROR-S) [46].

To predict pCR, we randomly split the MDACC225 [37,53] database into a training set (n = 150, ~2/3) and a testing set (n = 75, ~1/3), stratified by pCR and the clinical parameters HER2 and ER. HER2-positive patients who received trastuzumab (n = 4) and patients without complete clinical data (n = 1) were excluded (thus giving 225 patients). Using clinical variables (ER, histological grade, tumor size and HER2) and 318 modules (after excluding the Response_Predictor_MDACC [37,53], ROR-S [46], NKI 70-gene signature [3,4], the OncotypeDX RS [5], and the Rotterdam 76-gene signature [47]), we built pCR predictive models using clinical variables only, genomic variables only and a combination of both in the training set, and applied them in the testing set (n = 75) using a LASSO plus Logistic Regression approach. We then analyzed the receiver operating characteristic (ROC) curve for both the training and testing sets, and calculated the area under the ROC curve (AUC). In Additional File 2 we also calculated the AUC using the Response_Predictor_MDACC [37] module, which was trained and test in a subset of patients of the MDACC225 database [37]. All statistical analyses were performed with the use of the R software, version 2.9.0 http://www.r-project.org.

## Results

### Model Building and Risk Predictions

**Figure 1:**
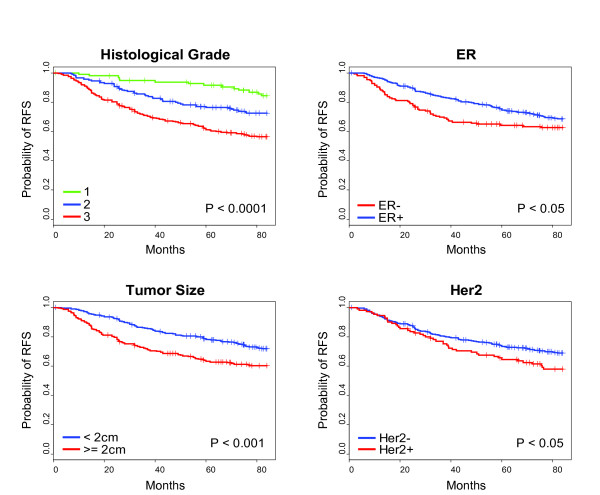
**Kaplan-Meier survival estimates of relapse-free survival (RFS) among 550 patients, according to tumor size, clinical estrogen receptor (ER) status, HER2 mRNA status, and histological grade**. P-values were obtained from the log-rank test, and (+) denotes observations that were censored owing to loss to follow-up or on the date of last contact.

In order to examine the potential of new prognostic models, a large and homogenously treated data set of breast cancer patients was created by combining together systematically untreated (i.e. no systemic adjuvant therapy) patients; the clinical and pathologic characteristics of the 550 patient dataset of node-negative, local therapy-only, breast cancer patients from the public domain are described in Table 1. The majority of patients had ER-positive (71.8%) and HER2-negative (80%) tumors, tumor size <2 cm (56.2%), and histological grade 1-2 (50.9%). The median follow-up of this population was 7.0 years (average 5.3 years). Tumor size, ER status, HER2 status, and grade were each prognostic for 7-year RFS (Figure 1), confirming that the combined dataset shows the expected outcomes for known biomarkers, and suggesting that the gene expression-based HER2 status designation (see Methods) was performing appropriately. No statistical significant differences for outcome were observed across the datasets that were combined together here (Additional File 2).

**Table 1:** Characteristics of the Combined Dataset.

				Training (~2/3)	Testing (~1/3)	
						
Characteristics		Total N	%	N	N	P-value*
Subjects		550		359	191	-

ER	+	395	71.8%	259	136	0.89
	-	155	28.2%	100	55	

Size	< 2 cm	309	56.2%	198	111	0.56
	≥ 2 cm	241	43.8%	161	80	

HER2*	+	110	20.0%	73	37	0.88
	-	440	80.0%	286	154	

Grade	1	98	17.8%	63	35	0.45
	2	182	33.1%	113	69	
	3	270	49.1%	183	87	

Published Dataset^	Ivshina	137	24.9%	89	48	1
	Loi	42	7.6%	28	14	
	NKI	141	25.6%	92	49	
	UNC	33	6.0%	22	11	
	Wang	197	35.8%	128	69	

Platform	Affymetrix	376	68.4%	245	131	0.99
	Agilent	174	31.6%	114	60	

Subtype (PAM50)	Luminal A	156	28.4%	98	58	0.92
	Luminal B	131	23.8%	85	46	
	HER2-enriched	83	15.1%	56	27	
	Basal-like	106	19.3%	72	34	
	Normal Breast-like	74	13.5%	48	26	

*HER2 status is based on ERBB2 mRNA levels. P-values have been calculated based on a Chi-square test.

^compiled from Ivshina et al., 2006; Loi et al., 2007; van de Vijver et al., 2002; Wang et al., 2005; http://www.ncbi.nlm.nih.gov/geo/query/acc.cgi?acc=GSE15393.

**Figure 2:**
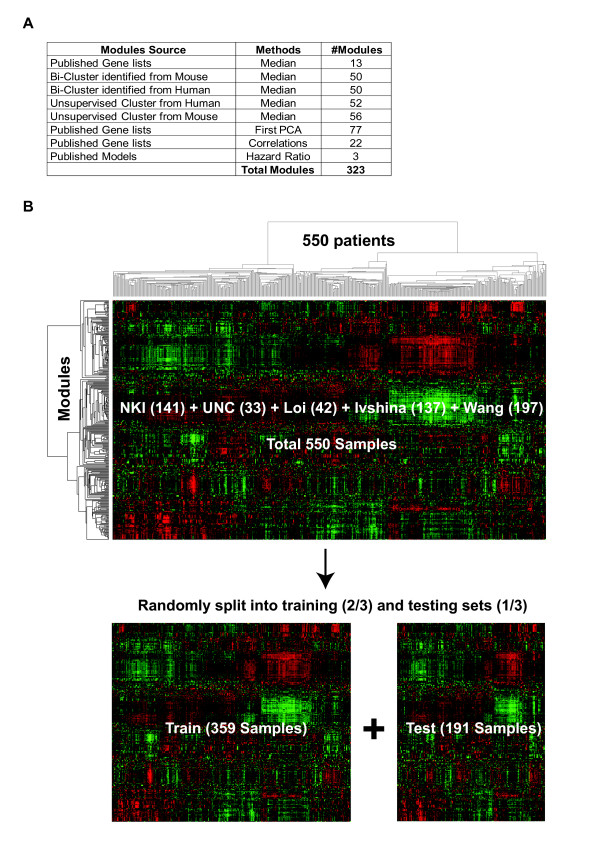
**Depiction of the combined breast tumor dataset**. (**A) **Table summarizing the different approaches used to obtain the various modules. PCA, principal component analysis. (**B) **Hierarchical cluster analysis of 323 gene expression modules (rows) across the microarray data of 550 node-negative breast cancer patients (columns). All samples were stratified by source, platform and clinical variables, and randomly split into a training (~2/3) and testing (~1/3) sets.

Along with clinical-pathological variables we applied 323 different modules to the combined dataset (Figure 2). Using a Cox proportional hazards approach with LASSO Regression [69], which is a method of model building that can handle large numbers of potentially co-linear variables, all patients and different patient subsets defined by clinical parameters or intrinsic molecular subtyping were tested for prognostic model building.

**Figure 3:**
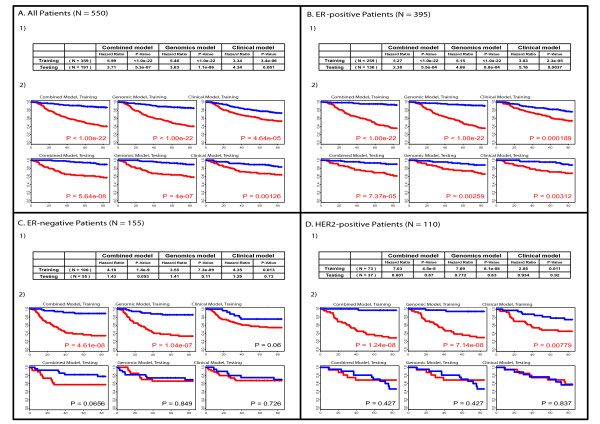
**Survival prediction analyses of the different Cox models**. (**A) **Models for all patients; (**B**) Models for ER-positive patients; (**C**) Model for ER-negative patients; (**D**) Models for HER2-positive patients. 1) Hazard ratio and p-value of the Cox proportional hazard model (Cox-model), for both the training and testing sets, respectively; 2) Kaplan-Meier survival estimates of relapse-free survival (RFS) among training and testing sets, respectively, according to each model. Patients were stratified into high-risk (red curve) and low-risk (blue curves) groups based on their respective risk score, which was defined as the natural logarithm of the hazard ratio. The chosen cut-off value for stratification into high and low-risk groups was zero. P-values were obtained from the log-rank test. + denotes observations that were censored owing to loss to follow-up or last contact.

Successful prognostic models were built for (a) all patients (Figure 3a) and (b) patients with ER-positive tumors (Figure 3b). Similar significant outcome predictions were also obtained for the cohort of patients defined as having Luminal tumors (i.e. Luminal A and B combined) and for ER-positive/HER2-negative patients (Additional File 2). Despite the fact that several modules were prognostic in univariate Cox proportional hazard analyses for either ER-negative or HER2-positive disease, no multivariate models could be built that held up in the both the training and testing sets for these patient subsets (Figure 3c-d). Interestingly, the combined model testing set for ER-negative disease approaches significance; however, there is little evidence of prognostic ability in HER2-positive disease. As expected, no prognostic models were obtained when we stratified patients based on the intrinsic subtype (Basal-like or HER2-enriched tumors), or based upon ER and HER2 status (ER-negative/HER2-negative, ER-negative/HER2-positive and ER-positive/HER2-positive tumors); it should be noted, however, that the sample size for some of the clinically defined patient subsets was small, which may have hindered successful model building.

### Selected Modules for Prognosis Predictions for All Patients

**Figure 4:**
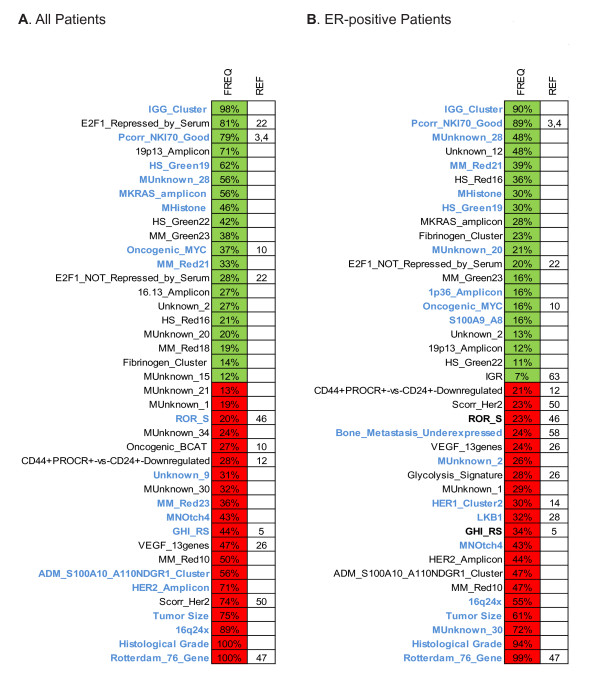
**Most frequently selected modules and clinical variables that build successful combined models for all patients (**A**) and ER-positive patients (**B**)**. Modules in blue identify those modules and/or clinical variables that were evaluated in the combined model in Fig. 2. Colored squares identify the modules and/or clinical variables association with either poor (red) or good (green) prognosis. Freq, frequency of selection of a particular module/clinical variable among 200 successful models; Ref, references of previously published modules.

In order to robustly identify prognostic variables, 200 rounds of training and testing were performed for each patient subset discussed above. Examination of the most frequently selected modules and clinical variables for the "all patients' group was revealing of the underlying biology (Figure 4a). Starting with all patients, the expression of several modules, in addition to tumor size and grade, was clearly associated with either a poor or good prognosis in the combined model. Included within the most frequently selected poor outcome modules were the Rotterdam 76-gene signature [47], the OncotypeDX RS (GHI_RS) [5], and the correlation to the HER2-enriched intrinsic subtype centroid (Scorr_Her2) [50]. In addition, other previously unpublished signatures derived from unsupervised hierarchical clustering analyses of human breast tumors and mouse mammary tumor datasets were also frequently selected to build prognostic models for all patients. Three of these poor prognostic modules were highly enriched with genes involved in cell cycle/proliferation (MM_Red10), vascular smooth muscle contraction (MUnknown_34), and mRNA processing/splicing (Unknown_9 and MUnknown_1); it is likely that our "undescribed" cell cycle/proliferation module is merely reflecting many previously described proliferation signatures [71], which is a known and powerful predictor of outcomes for node-negative breast cancer patients.

Increased expression of other unpublished modules was associated with good outcome. Several of these were highly enriched with genes involved in immune response (IGG_Cluster; and HS_Red16), extracellular space (HS_Green19 and Fibrinogen_Cluster), transcription (MHistone) and cation transport (MM_Red21). Of note, the IGG_Cluster (Immunoglobulin) 14-gene module, which was identified by unsupervised clustering of human breast tumors, was selected in 196 of 200 combined models (98%). Consistent with our findings, data from a recent report suggests that, besides proliferation, the combination of immunity and RNA splicing processes may have a high prognostic impact in breast cancer [43].

Among the rest of the modules that frequently comprised the combined model for all patients, it is interesting to note that two of them were derived from a single study that evaluated the transcription factor E2F1-dependent gene expression program [22], two others were derived from predictions of β-catenin and MYC pathway deregulation in cancer cell lines [10], and another one was likely tracking stem cell-like biological processes [12].

### Selected Modules for Outcome Prediction for ER-positive Tumors

The majority of combined models built for ER-positive patients included grade, tumor size, and various modules whose expression was associated with either poor or good prognosis (Figure 4b). Among the 40 most frequently selected modules, 31 (77.5%) were previously selected to build combined models for all patients, suggesting that outcome predictions for all patients is being largely driven by the ER-positive patient subset. Importantly, the Rotterdam 76-gene signature [47], the NKI 70-gene signature [3,4] and the OncotypeDX RS [5], which were specifically designed to risk stratify early-stage ER-positive breast cancer, were found among the top most frequently selected modules that build the final combined model. In addition, high expression of the previously described IGG_Cluster 14-gene immune response module was also found highly associated with good prognosis in this clinically identifiable breast cancer subtype.

We also repeated the analysis without the four best-known prognostic profiles (i.e. the Rotterdam 76-gene index [47], OncotypeDX RS [5], NKI-70-gene signature [3,4] and ROR-S [46]); in this secondary analysis, 31 of the 40 most frequently selected modules that built the previous combined models for ER-positive patients were again selected, including tumor size and grade (Additional File 2). However, two previously unobserved and highly selected modules were identified that were the HS_Red23 module that was present in 143 of 200 models (71.5%, which mainly tracks cell cycle/proliferation), and the correlation to the Luminal A intrinsic breast cancer subtype centroid (Scorr_LumA), which was present in 177 of 200 models (88.5%) and, as expected, was associated with good outcome. Again, no prognostic models could be built for ER-negative, or HER2-positive patients, that were successful on both the training and test sets.

### Performance of the Clinical, Genomic and Combined Models

**Figure 5:**
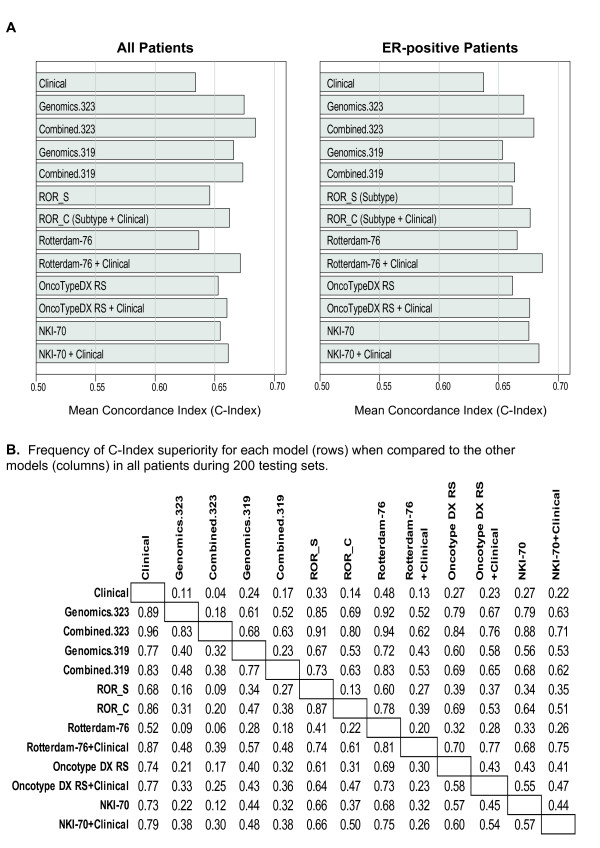
**C-Index evaluations of the various models analyzed**. (**A**) Performance of clinical, genomic and combined models in the testing sets of all patients and ER-positive patients. Each patient subset was randomly split into a training set (~2/3 of cases) and a testing set (~1/3 of cases). We then used the model built from the training set to calculate the C-index of the testing set. We repeated this procedure 200 times and then calculated the mean of the C-index for each model. The performance of established prognostic predictors (OncoTypeDX RS, NKI 70-gene signature, 76-gene Rotterdam index, the risk of relapse based on intrinsic subtyping [ROR_S]) with or without the addition of clinical variables was also estimated. (**B**) Frequency of superiority of the C-Index for each model (rows) when compared to the other models (columns) in 200 testing sets of all patients. Each row represents a model, which is then compared to all other models/columns, where a higher number indicates that the row model was superior to the model in the column that fraction of the 200 times tested.

The prognostic ability of clinical variables, genomic variables and a combination of both was further characterized by calculating the concordance index (C-index) [70] in the testing sets of (a) all patients and (b) ER-positive patients after 200 randomizations into training and testing sets (Figure 5A); we acknowledge that these rounds of training and testing show dependency in that samples from one round of training will become test samples in other rounds, but given the limitations of this data set in terms of size and diversity, we felt this to be a good means of assessing the relative accuracy of a diverse set of genomic and pathological predictors. In both cohorts (all patients and ER+ only), there was an improvement in prediction with the use of genomics relative to the model of clinical variables only (Genomics.323 vs. Clinical). More importantly, a combination of clinical and genomic variables (Combined.323) showed an improvement over either predictor alone in both patient cohorts (Combined.323 vs. Genomics.323) even when the prognostic signatures NKI 70-gene signature [3,4], the OncotypeDX RS [5], the Rotterdam 76-gene signature [47] and the recently described Risk of Relapse score (ROR) based on the intrinsic subtypes (ROR-S) [46] were removed from the analysis (i.e. Combined.319). Indeed, the C-Index of the Combined.319 model was superior to the Genomics.319 model in 77% of the 200 rounds of testing when using all patients (Figure 5B), as were most other instances of a combined model versus the genomic only version.

The performance of the clinical, genomic and combined models in all patients and ER-positive patients was also compared to the other known 4 prognostic signatures with and without the addition of clinical variables (Figure 5A). It should be noted that a subset of patients in this combined dataset were part of the training dataset used to derive all these prognostic signatures, except for the OncoTypeDX RS [5]. Nonetheless, as shown in Figure 5A, the Combined.323 models modestly improved the concordance index in all patients (i.e. 0.6844 vs. 0.6622 ROR-C) or performed similarly in ER-positive patients (i.e. 0.6798 vs. 0.6764 ROR-C) as did the other known prognostic predictors when combined with clinical variables. This data suggests that combining clinical variables with a single genomic module is better than either one alone, and that combining clinical variables with a single module such as the OncoTypeDX RS (21 genes) can perform similarly as a combined model that includes hundreds of signatures.

### Prediction of Response to Neoadjuvant Chemotherapy

**Figure 6:**
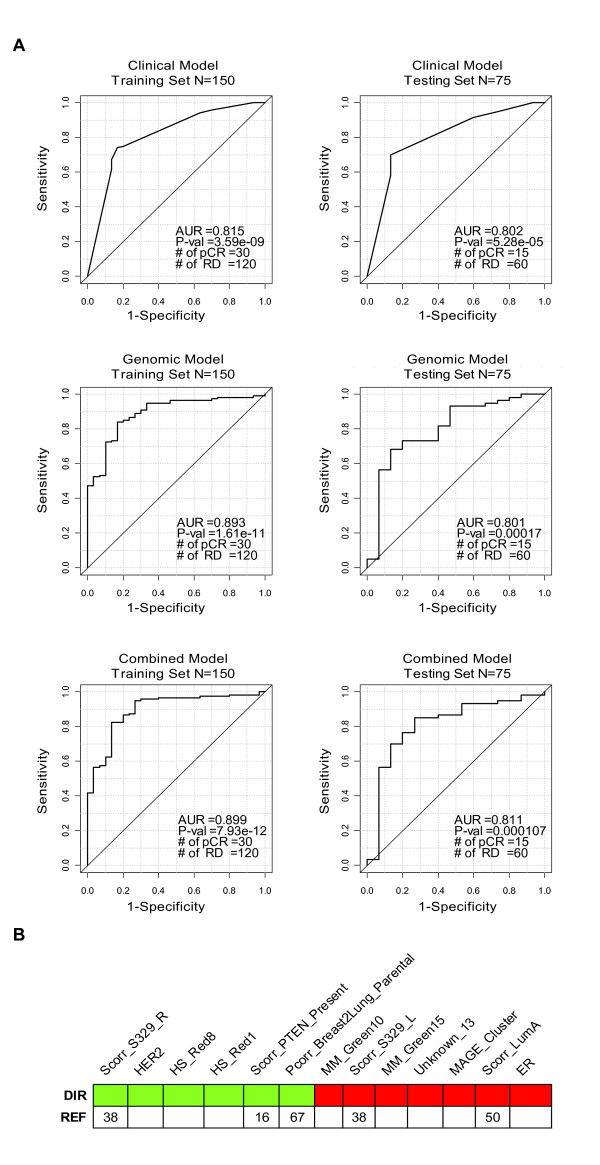
**Integration of clinical and genomic variables to predict pathological complete response (pCR) after anthracycline/taxane-based chemotherapy using Popovici et al. dataset (n = 225)**. (**A**) Area under the receiver operating characteristic curve (AUC) for clinical, genomic and combined models in the training and testing sets. (**B**) Modules and clinical variables that built the combined model evaluated in section (A). Colored squares identify the modules and/or clinical variables association with pCR (red) or non-pCR (green), respectively. Ref, references of previously published modules. Note: Response_Predictor_MDACC, OncoTypeDX RS, NKI 70-gene signature, 76-gene Rotterdam index and ROR-S have been removed for this analysis.

To show the potential application of this combined approach in predicting treatment response, we applied the different genomic modules, along with clinical variables (tumor size, HER2, ER, and histological grade), to 225 patients with breast cancer treated with a taxane and anthracycline containing neoadjuvant chemotherapy regimen [37,53] (i.e. T/FAC). We were able to build statistically significant combined models for pCR prediction using 150 patients (training data), and obtained an average accuracy of 81% in the testing set (n = 75) (Figure 6a). Among the modules and clinical variables selected to build the combined model, HER2 status, a module that tracks immune system response, cell proliferation (HS_Red1), and a module that tracks cell-adhesion/differentiation (HS_Red8) were associated with pCR (Figure 6b), while ER status and correlation to the Luminal A intrinsic breast cancer subtype centroid (Scorr_LumA) were associated with non-pCR. Interestingly, two previously published modules of non-taxane chemotherapy responsiveness (Scorr_S329_R and Scorr_329_L)[38] were selected in the combined model. Once again, the combined model outperformed the clinical or genomics models only as assessed by AUC values.

## Discussion

Both genomic biomarkers and clinical parameters provide prognostic powers [2,7,41-43], however, very few studies have attempted to combine these disparate data types into a single statistical model [45,46]. To address this challenge we undertook a comprehensive evaluation of the prognostic ability of hundreds of genomic modules in combination with clinical variables, and evaluations of well known genomic predictors alone and in combination with clinical variables. An important caveat to these analyses is that for all published predictors tested here, each lost genes due to the data set combining, and a common data normalization method was used, and thus we caution against interpreting these data to mean that one specific predictor is better than another. Instead, these exploratory and comparative analyses have highlighted important concepts that should be the foundation for future studies. Specifically, we show that hundreds of genomic signatures are only slightly better than a few well developed signatures, and that the integration of gene expression signatures and clinical-pathological factors can improve prognostication in patients with lymph node-negative ER-positive breast cancers. However, for ER-negative and HER2-positive breast cancer patients, other variables beyond gene expression and clinical-pathological variables will be needed to build robust prognostic models. Alternatively, ER-negative or HER2-positive patients may have a stereotypical poor prognosis, and thus, building predictive models of therapy responsiveness would be much more relevant for these two disease subtypes.

While proliferation-related genes are an important part of many prognostic gene sets, these signatures show no prognostic value when the analysis is limited to ER-negative patients, as has been shown before [72]. The majority of established prognostic signatures were mainly designed for ER-positive breast cancer patients. The NKI 70-gene signature [3,4] included a subgroup of patients with ER-negative and HER2-positive disease in their validation studies, however, this and other signatures developed using all patients are heavily influenced by proliferation and ER-related genes, and therefore, classify almost all of these highly proliferative ER-negative tumors into the high risk category [6]. Despite including signatures that track many other distinct biological processes, no modules or combination of modules were able to build robust models for ER-negative, or HER2-positive patients. However, it is interesting to note that in the ER-negative population tested here, the expression of the IGG_Cluster immune response module was associated with a favorable outcome in univariate analyses, and this module was included, along with gene lists that track apoptosis and developmental processes, in the combined model for the ER-negative training set (data not shown); however, these predictors were not statistically significant in the test set. These negative results should be interpreted with caution as failed predictions may be due to the technical limitations of this dataset, however, these results do suggest that studies focusing on the specific biological role of immune cells on tumor progression and/or response to treatment may be warranted in ER-negative tumors. Concordant with our findings, previous studies [31,32,73,74] have suggested that the absence of immune response related-genes might be associated with the development of distant metastases and therefore poor outcome in HER2-positive and ER-negative breast cancer.

Although the gene lists of the majority of "selected" modules/signatures were largely non-overlapping, significant agreement in outcome predictions for individual patients was observed, which confirms and extends our previous observations [6] that different modules can reflect similar biological processes despite small overlaps in gene identity. For example, many signatures that are likely tracking proliferation were commonly selected including the OncotypeDX RS [5], a signature of chromosomal instability (CIN70) [21], and two signatures that recapitulates the loss of the retinoblastoma gene [17,18], all of which showed a cluster correlation of ~0.8 (Additional File 2). Conversely, other highly correlated signatures showed a high negative correlation to these proliferation-related modules, which is the case for the estrogen and GATA3-regulated signatures [9,40], immune response signatures [27,31], the NKI good prognostic signature [3,4], signatures that track cell differentiation [30], and lack of response to chemotherapy [37].

While we have recapitulated known biology and made novel observations that are relevant to breast cancer patient management, there are limitations to this study. First, the sample size of the entire data set was sufficient to build strong predictors when considering all patients and for ER-positive patients. However, when conducting stratified analyses, sample size significantly decreased in some cases (i.e. only 106 Basal-like patients), thus limiting power for those subsets. This represents an alternative explanation for the failure to identify prognostic signatures within those groups, although it is quite possible that almost all Basal-like patients do have an inherently poor prognosis in the untreated setting and that no identifiable "good prognosis signature" exists for Basal-like tumors. Second, a challenge of this study was the implementation of many published predictors onto a common dataset, many of which used unique statistical methods; we strove to implement each predictor as published, however, almost all predictors "lost" genes due to combining data across microarray platforms, and thus, almost all predictors differed somewhat from their original specification. Lastly, this dataset is comprised primarily of tissue bank samples and therefore factors such as patient selection may differ from what is observed in a true population-based sampling of incident cases.

Despite these caveats, our analyses did identify robust predictors for all patients and for ER-positive patients, and confirmed the prognostic abilities of many previously published signatures. In addition, we demonstrated that models built using clinical variables can be improved with genomic information, and showed that the best models are a combination of genomic and clinical variables. Another implication of these data is that prognostication for breast cancer patients is possibly only relevant for ER-positive/Luminal patients; it does not adequately prognosticate in ER-negative or HER2-positive breast cancers. Similarly, a recent study suggests a markedly poorer prognosis in even the smallest node-negative HER2-positive tumors [75]. Additional data, or methods, are still needed for prognostication in these patients.

Clearly models focused on predicting chemotherapy responsiveness would be highly relevant to ER-negative tumors and breast cancer patients in general. To address this predictive need, we successfully applied our model building approach on a set of 225 T/FAC treated patients and achieved AUC values of ~0.8. However, we were unable to test if pCR predictors are of great value for HER2-positive and Basal-like patients survival outcomes due to the lack of a dataset that included outcomes, neoadjuvant response and microarray data.

Lastly, some individual models, like the NKI 70-gene signature [3,4], the OncotypeDX RS [5], the 76-gene Rotterdam index [47] and the ROR-C model [46], achieved comparable predictive abilities when compared to the full new model containing ~20 modules composed of thousands of genes and multiple clinical variables; we acknowledge that for the majority of these predictors (excluding the OncotypeDX RS), the dataset used here could be considered to be a training data set, which likely over estimates their true prognostic powers. However, the newly developed combined models did use training and testing sets and barely outperformed the aforementioned predictors, which suggests that not much more prognostic powers are to be had by including hundreds of signatures beyond the powers contained within a well developed individual signature when combined with the clinical variables. Ultimately, we envision that additional data types like germline genotypes, splicing information, microRNA profiles, DNA Copy number changes, phospho-proteomic patterns and gene mutation information will lead to significant improvements to the existing models, and in theory our model building approach can incorporate these disparate data types.

## Conclusions

In this study, we have identified significant prognostic models using clinical-pathological variables and 323 gene expression "modules" in patients with lymph node-negative ER-positive breast cancer. Specifically, we show that 1) the clinical variables alone build the least accurate models, 2) genomic models alone are better than clinical variables alone, and 3) a combined genomic and clinical model is best, and thus, would be the most helpful in the process of adjuvant decision-making for node-negative ER-positive breast cancers. These findings reinforce that clinical information still plays a key role for prognostication in node-negative breast cancer (especially in ER-positive disease), but also that genomic variables provide important information not provided by the classical clinical variables. Interestingly, we also found that single module/signatures built for prognosis (i.e. ROR-S, NKI 70-gene, OncotypeDX RS, Rotterdam 76-gene index) can perform nearly as well as a combination of hundreds of signatures. Finally, although huge advancements in prognostic models for breast cancer are unlikely given the amount of work that has already gone into biomarkers for breast cancer, we feel that our advances are significant, even if the magnitude of the effect is not large.

## Competing interests

CMP is the co-founder of University Genomics and is a major stock holder of University Genomics and Bioclassifier LLC. JSP and CMP are also listed as inventors on patents filed pertaining to the PAM50 and ROR score assays.

## Authors' contributions

CF analyzed the data and performed the statistical analysis. AP analyzed the data and wrote the manuscript. JSP and YL provided technical support. CMP, LAC and MAT participated in the design of the study and helped to draft the manuscript. All authors read and approved the final manuscript.

## Pre-publication history

The pre-publication history for this paper can be accessed here:

http://www.biomedcentral.com/1755-8794/4/3/prepub

## Supplementary Material

Additional file 1 **Supplemental materials and methods**. This file contains all the gene lists and methods used for the 323 modules evaluated in this article. Click here for file

Additional file 2 **Supplemental data**. This file contains additional analyses and results. Click here for file
