# Supplementary material for: Building prognostic models for breast cancer patients using clinical variables and hundreds of gene expression signatures
Source: BMC Med Genomics. 2011 Jan 9;4:3. doi: 10.1186/1755-8794-4-3 (PMC3025826; doi:10.1186/1755-8794-4-3)

SUPPLEMENTAL DATA

to

“***Building Prognostic Models for Breast Cancer Patients Using Clinical Variables and Hundreds of Gene Expression Signatures***”

by

*Cheng Fan, Aleix Prat, Joel S. Parker, Yufeng Liu, Lisa A. Carey, Melissa A. Troester and Charles M. Perou*

## Supplementary Tables and Figures

**Supplemental Table 1.** Summary of the combined models built from expression and clinical variables in the different patient cohorts.

**Supplemental Figure 1.** Kaplan-Meier survival estimates of relapse-free survival among 550 patients, according to the data set evaluated.

**Supplemental Figure 2a-c.** Unsupervised hierarchical cluster analysis of 323 gene expression modules (rows) across the microarray data of 550 node-negative breast cancer patients (columns). Exact localization of modules is shown on the right side of figures 2a, 2b and 2c.

**Supplemental Figure 3**. Survival prediction analyses of the different models under evaluation from a total of 319 modules (which excludes the known modules that were trained on patient prognosis). (**A**) Models for all patients; (**B**) Models for ER-positive patients; (**C**) Models for ER-negative patients; (**D**) Models for HER2-positive patients. 1) Hazard ratio and p-value of the Cox proportional hazard model (Cox-model), for both the training and testing sets, respectively; 2)

Kaplan–Meier survival estimates of relapse-free survival among training set and testing set, according to each model. Patients were stratified into high-risk (red curve) and low-risk (blue curves) groups based on their respective risk score, which was defined as the natural logarithm of the hazard ratio. The chosen cut-off value for stratification into high and low-risk groups was zero. P-values were obtained from the log-rank test. + denotes observations that were censored owing to loss to follow-up or on the date of last contact.

**Supplemental Figure 4**. Significant prognostic combined models built for all patients (**A**) and ER-positive patients (**B**) from 319 gene expression modules (which excludes the known modules that were trained on patient prognosis). Modules in blue identify those modules and/or clinical variables that were evaluated in the combined model in Supplemental Figure 3. Colored squares identify the modules and/or clinical variables association with poor (red) or good (green) prognosis, respectively. Freq, frequency of selection of a particular module/clinical variable among 200 models; Ref, references of previously published modules.

**Supplemental Figure 5**. Performance of the MDACC module for predicting pathological complete response (pCR) after anthracycline/taxane-based chemotherapy using Popovici et al. dataset (n=225).

**Supplemental Table 1**. Summary of the Combined Models Built from Expression and Clinical Variables in the Different Patient Cohorts.

| Patient Cohort | Train | Test | Train | | Test | |
|---|---|---|---|---|---|---|
| | | | Cox HR* | P-value | Cox HR* | P-value |
| **All** | **359** | **191** | **5.99** | **<0.0001** | **3.71** | **<0.0001** |
| **ER-positive (ER+)** | **259** | **136** | **5.27** | **<0.0001** | **2.38** | **<0.001** |
| **ER+/HER2-negative** | **232** | **118** | **6.78** | **<0.0001** | **14.7** | **<0.001** |
| ER-negative | 100 | 55 | 4.18 | <0.0001 | 1.43 | 0.093 |
| HER2-positive | 73 | 37 | 7.03 | <0.0001 | 0.801 | 0.670 |
| **Luminal** | **183** | **104** | **4.67** | **<0.0001** | **2.33** | **<0.005** |
| Luminal A | 98 | 58 | 7.06 | <0.0001 | 0.996 | 0.99 |
| Luminal B | 85 | 46 | 11.4 | <0.0001 | 7.93 | 0.08 |
| Basal-like | 72 | 34 | 20.4 | <0.0001 | 2.26 | 0.59 |
| HER2-enriched | 56 | 27 | NA | NA | NA | NA |

*Cox HR, hazard ratio for RFS. NA, a Cox proportional hazard model could not be built.

**Supplemental Figure 1**

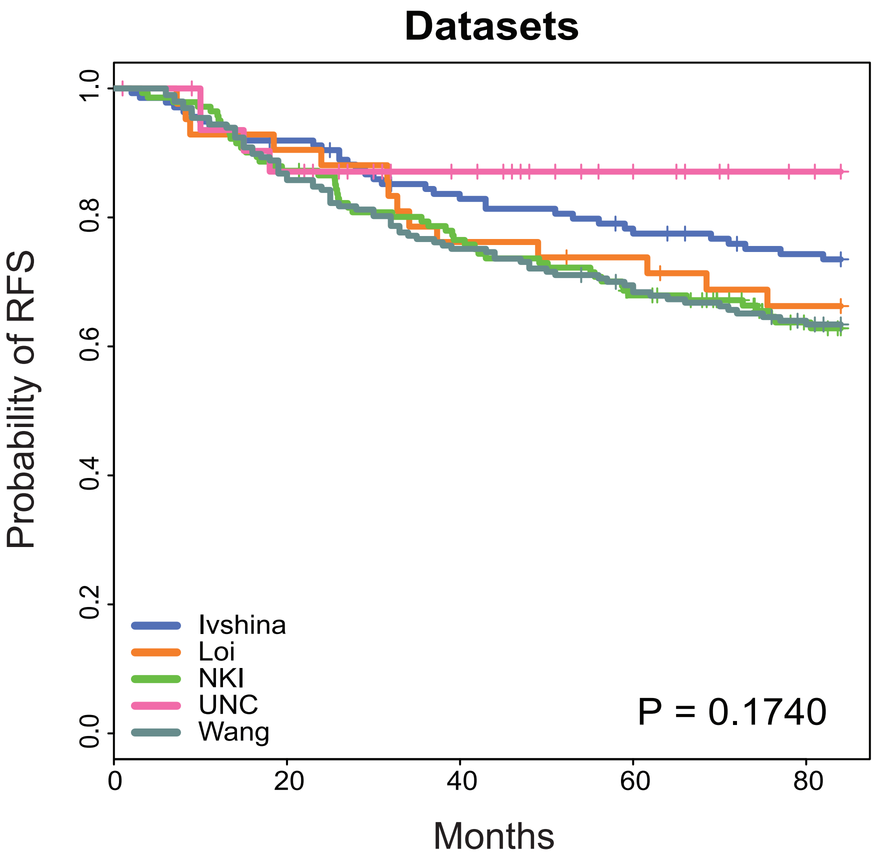

# Supplemental Figure 2a

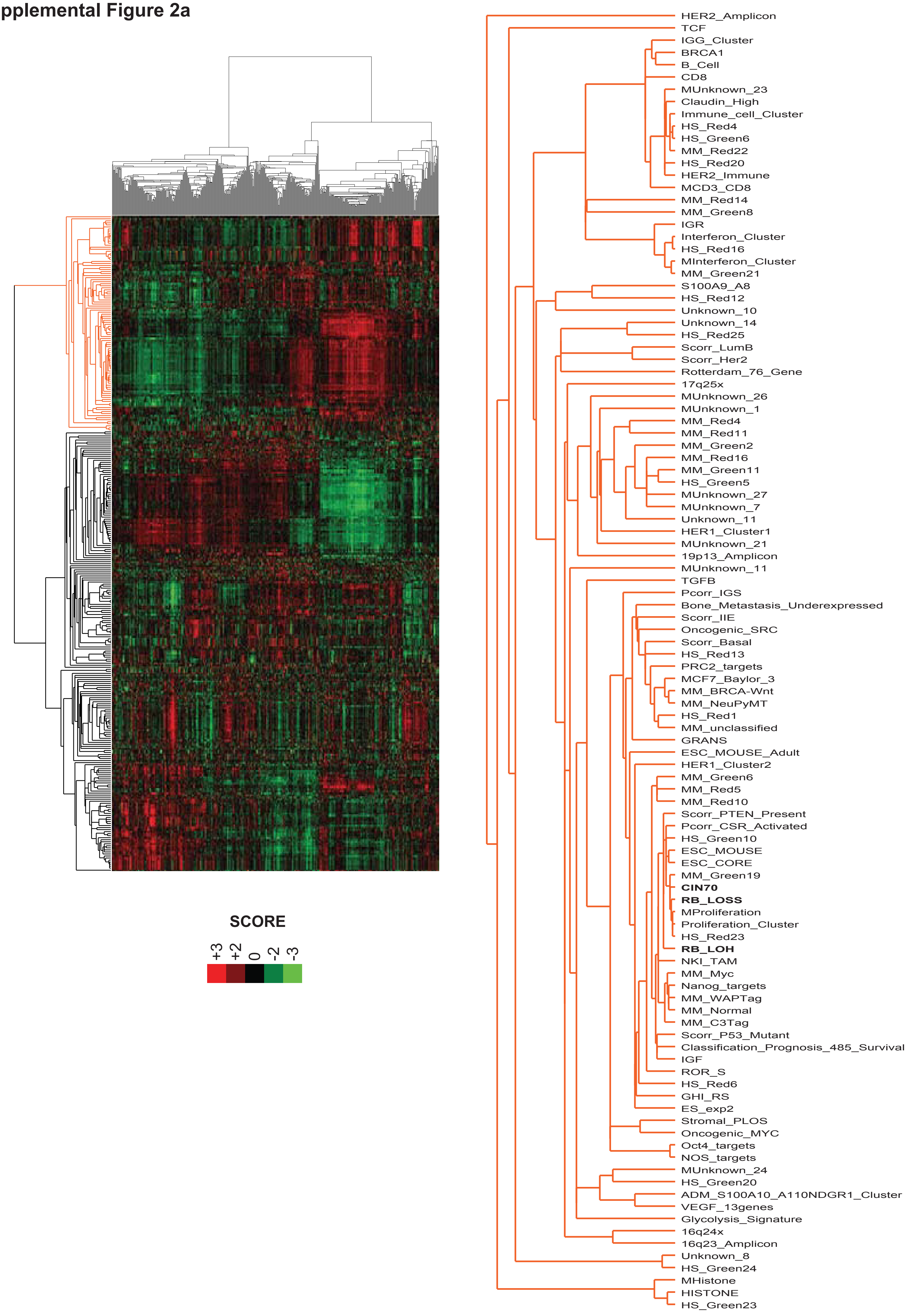

**Supplemental Figure 2b**

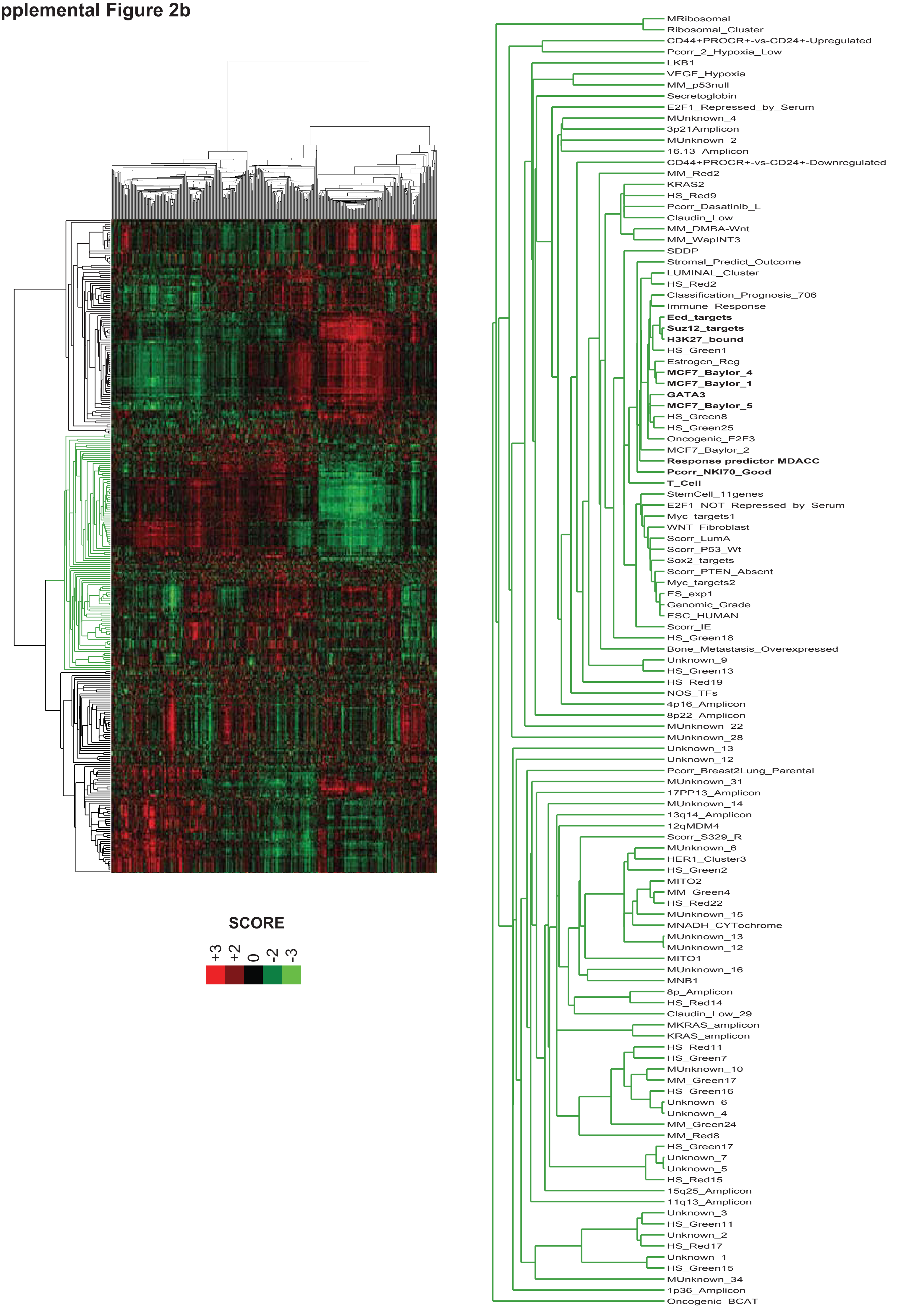

## Supplemental Figure 2c

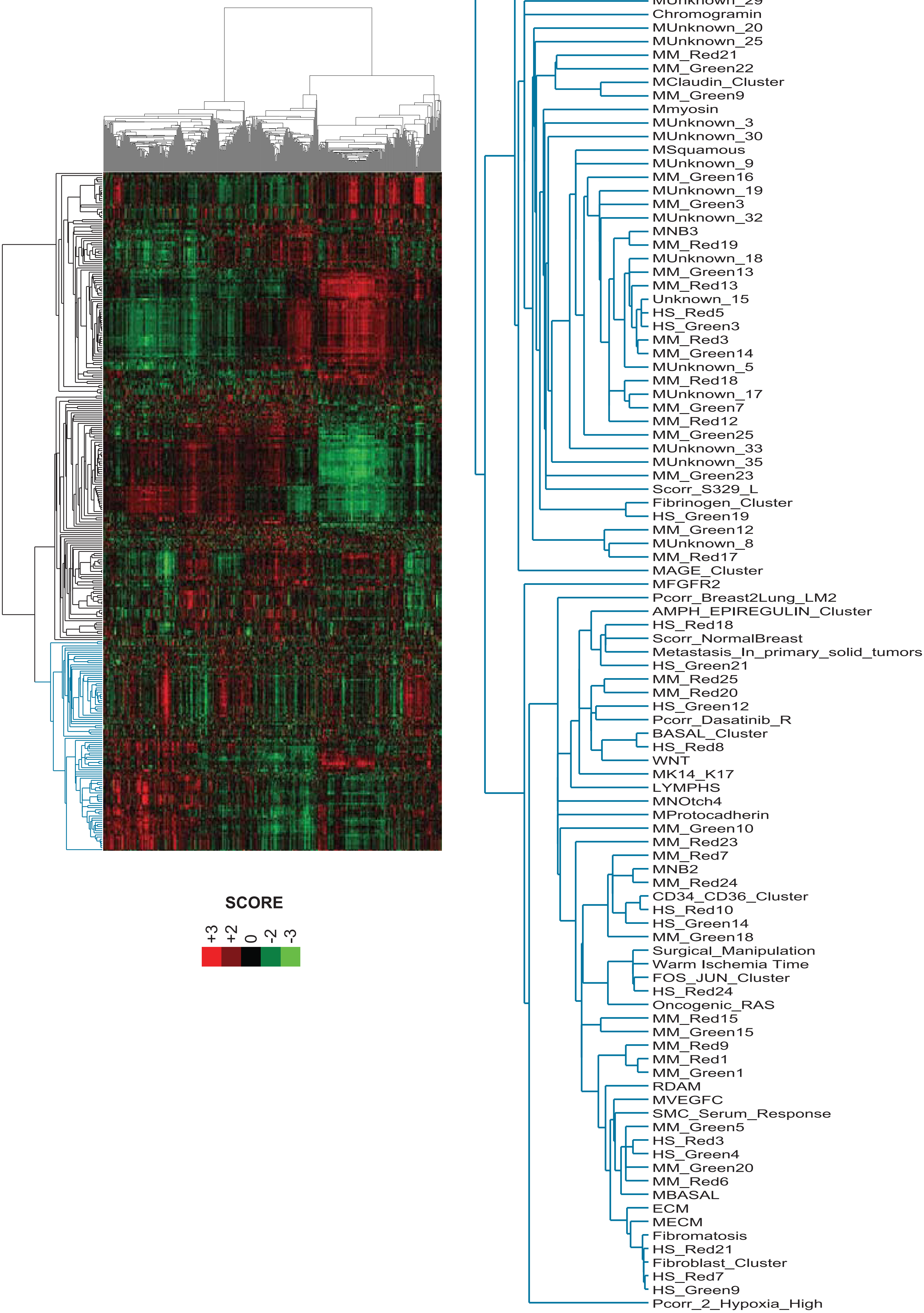

# Supplemental Figure 3

## A. All Patients (N = 550)

1)

| | | Combined model | | Genomics model | | Clinical model | |
|---|---|---|---|---|---|---|---|
| | | Hazard Ratio | P-Value | Hazard Ratio | P-Value | Hazard Ratio | P-Value |
| Training | ( N = 359 ) | 6.28 | <1.0e-22 | 6.7 | <1.0e-22 | 3.34 | 3.4e-06 |
| Testing | ( N = 191 ) | 3.47 | 9.3e-06 | 4.48 | 9.1e-06 | 4.34 | 0.001 |

2)

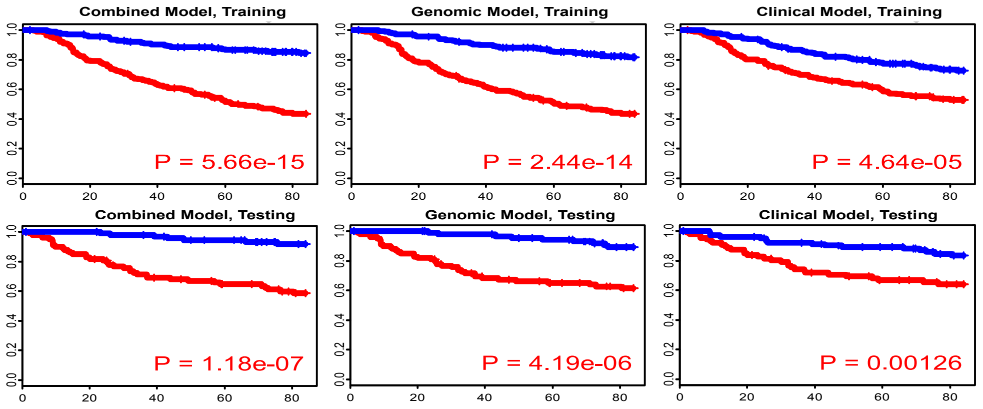


## B. ER-positive Patients (N = 395)

1)

| | | Combined model | | Genomics model | | Clinical model | |
|---|---|---|---|---|---|---|---|
| | | Hazard Ratio | P-Value | Hazard Ratio | P-Value | Hazard Ratio | P-Value |
| Training | ( N = 259 ) | 5.45 | <1.0e-22 | 5.8 | <1.0e-22 | 3.83 | 2.3e-05 |
| Testing | ( N = 136 ) | 1.95 | 0.0097 | 2.15 | 0.011 | 5.16 | 0.0037 |

2)

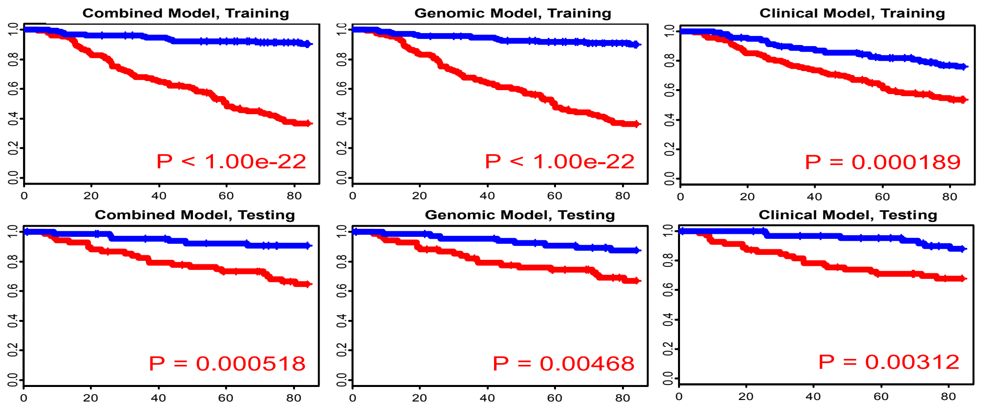


## C. ER-negative Patients (N = 155)

1)

| | | Combined model | | Genomics model | | Clinical model | |
|---|---|---|---|---|---|---|---|
| | | Hazard Ratio | P-Value | Hazard Ratio | P-Value | Hazard Ratio | P-Value |
| Training | ( N = 100 ) | 3.92 | 5.5e-9 | 3.52 | 5.5e-08 | 4.35 | 0.013 |
| Testing | ( N = 55 ) | 1.51 | 0.073 | 1.47 | 0.096 | 1.29 | 0.73 |

2)

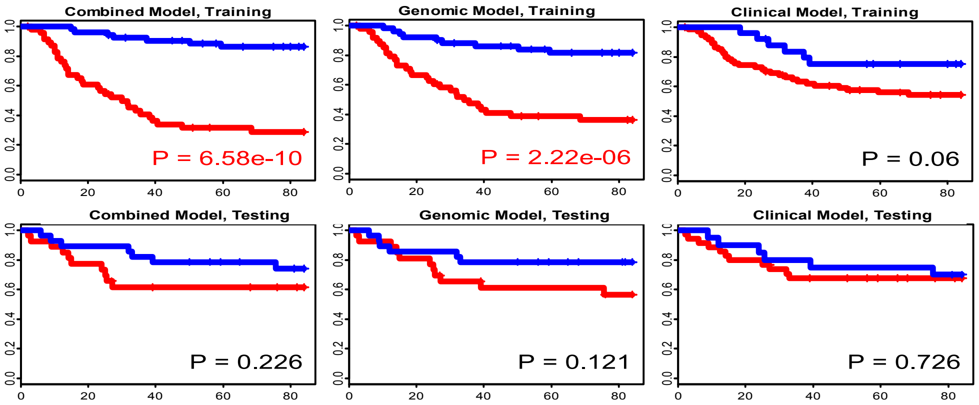


## D. HER2-positive Patients (N = 110)

1)

| | | Combined model | | Genomics model | | Clinical model | |
|---|---|---|---|---|---|---|---|
| | | Hazard Ratio | P-Value | Hazard Ratio | P-Value | Hazard Ratio | P-Value |
| Training | ( N = 73 ) | 7.54 | 7.6e-8 | 7.71 | 8.2e-08 | 2.85 | 0.011 |
| Testing | ( N = 37 ) | 0.777 | 0.64 | 0.77 | 0.63 | 0.934 | 0.92 |

2)

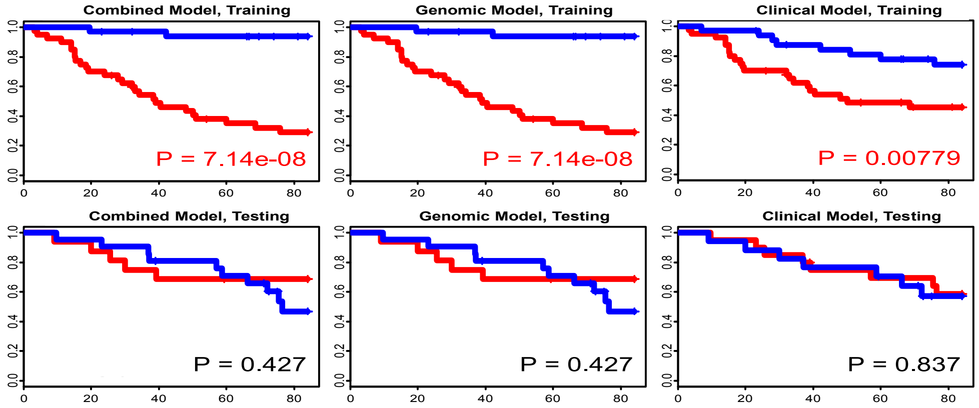

**Supplemental Figure 4**

**A.** All Patients

| | FREQ | REF |
|---|---|---|
| IGG_Cluster | 99% | |
| E2F1_Repressed_by_Serum | 86% | 22 |
| 19p13_Amplicon | 68% | |
| HS_Green19 | 62% | |
| MUnknown_28 | 58% | |
| E2F1_NOT_Repressed_by_Serum | 49% | 22 |
| HS_Green22 | 47% | |
| MM_Red21 | 47% | |
| MKRAS_amplicon | 46% | |
| MM_Green23 | 43% | |
| MNB1 | 25% | |
| 8p22_Amplicon | 21% | |
| MUnknown_20 | 21% | |
| MHistone | 20% | |
| Scorr_LumA | 19% | 50 |
| MM_Red18 | 19% | |
| Unknown_2 | 19% | |
| Oncogenic_MYC | 18% | 10 |
| Scorr_P53_Wt | 17% | 11 |
| MUnknown_15 | 17% | |
| ESC_CORE | 8% | 29 |
| HS_Red25 | 13% | |
| Pcorr_IGS | 16% | 68 |
| MM_p53null | 20% | 13 |
| NKI_TAM | 20% | 20 |
| MM_Red23 | 20% | |
| MUnknown_1 | 24% | |
| MUnknown_30 | 28% | |
| MNOtch4 | 30% | |
| Oncogenic_BCAT | 31% | 10 |
| CD44+PROCR+-vs-CD24+-Downregulated | 33% | 12 |
| HS_Red23 | 37% | |
| ADM_S100A10_A110NDGR1_Cluster | 53% | |
| MM_Red10 | 55% | |
| HER2_Amplicon | 59% | |
| VEGF_13genes | 61% | 26 |
| Tumor Size | 66% | |
| Scorr_Her2 | 88% | 50 |
| 16q24x | 97% | |
| Histological Grade | 98% | |

**B.** ER-positive Patients

| | FREQ | REF |
|---|---|---|
| IGG_Cluster | 90% | |
| Scorr_LumA | 89% | 50 |
| Unknown_12 | 48% | |
| E2F1_NOT_Repressed_by_Serum | 48% | 22 |
| MUnknown_28 | 39% | |
| MM_Red21 | 36% | |
| MNB1 | 30% | |
| HS_Red16 | 30% | |
| MHistone | 28% | |
| HS_Green19 | 23% | |
| 19p13_Amplicon | 21% | |
| 1p36_Amplicon | 20% | |
| MM_Green23 | 16% | |
| Scorr_IE | 16% | 9 |
| MKRAS_amplicon | 16% | |
| HS_Green22 | 16% | |
| Oncogenic_MYC | 13% | 10 |
| StemCell_11genes | 12% | 60 |
| Fibrinogen_Cluster | 11% | |
| Response predictor MDACC | 7% | 37 |
| HER2_Amplicon | 21% | |
| TGFB | 23% | 12 |
| LKB1 | 23% | 28 |
| Scorr_Her2 | 24% | 50 |
| MM_p53null | 24% | 11 |
| HS_Red25 | 26% | |
| CD44+PROCR+-vs-CD24+-Downregulated | 28% | 12 |
| MUnknown_1 | 29% | |
| ADM_S100A10_A110NDGR1_Cluster | 30% | |
| VEGF_13genes | 32% | 26 |
| MNOtch4 | 34% | |
| MM_Red10 | 43% | |
| Glycolysis_Signature | 44% | 26 |
| Tumor Size | 47% | |
| Bone_Metastasis_Underexpressed | 47% | 58 |
| HER1_Cluster2 | 55% | 14 |
| 16q24x | 61% | |
| HS_Red23 | 72% | |
| MUnknown_30 | 94% | |
| Histological Grade | 99% | |

## Supplemental Figure 5

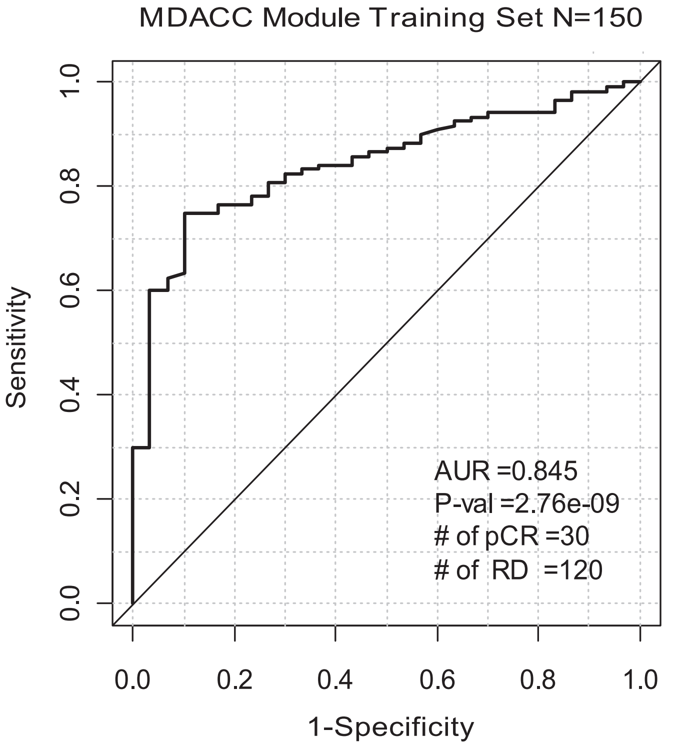


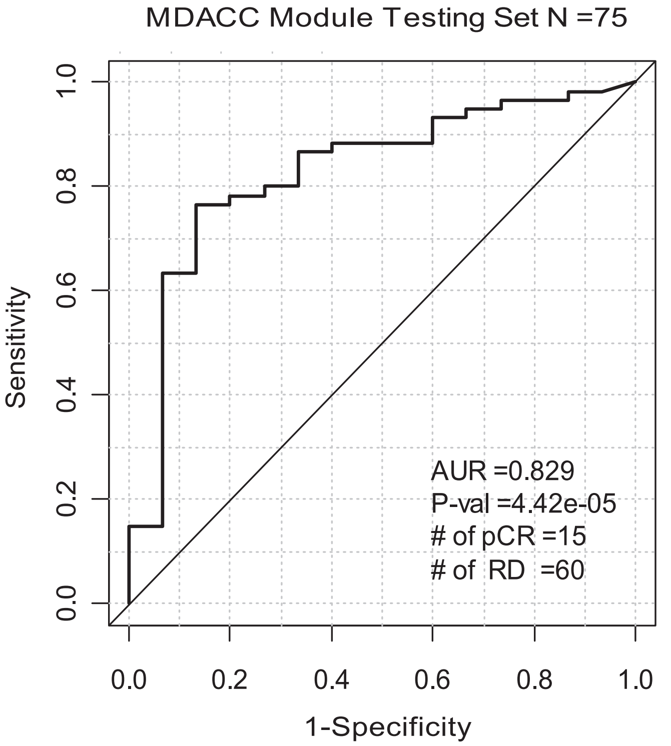

Supplement: Additional file 2 — Supplemental data. This file contains additional analyses and results. [file 1755-8794-4-3-S2.PDF]
